# Association of weight misperception with weight loss in a diabetes prevention program

**DOI:** 10.1186/1471-2458-14-93

**Published:** 2014-01-30

**Authors:** Andrea L Hernan, Vincent L Versace, Tiina Laatikainen, Erkki Vartiainen, Edward D Janus, James A Dunbar

**Affiliations:** 1 Greater Green Triangle University Department of Rural Health, Flinders University and Deakin University, PO Box 423, Warrnambool, VIC 3280, Australia; 2 Institute of Public Health and Clinical Nutrition, University of Eastern Finland, PO Box 1627, Kuopio 70211, Finland; 3 Hospital District of North Karelia, Tikkamäentie 16, Joensuu 80210, Finland; 4 National Institute for Health and Welfare, Mannerheimintie 166, Helsinki 00300, Finland; 5 Department of Medicine, North West Academic Centre, The University of Melbourne, Western Hospital, Footscray, VIC 3011, Australia

**Keywords:** Weight misperception, Weight loss, Diabetes prevention, Risk screening, Risk perception

## Abstract

**Background:**

Weight misperception may have an impact on perceived risk and susceptibility for chronic diseases. Little has been reported on the long term effects of this misperception in chronic disease interventions, particularly in field of diabetes prevention. The aim of this study was to investigate the relationship between weight misperception and weight loss during a diabetes prevention project conducted in south-east Australia with individuals at moderate to high risk of developing diabetes.

**Methods:**

A total of n=251 at risk individuals provided self-reported weight during recruitment from 2004-2006. Objectively measured weight was assessed at baseline (0-21 days after recruitment), and subsequently at three months and 12 months after the intervention. Differences between self-reported and actual weight status are presented as percentages. Linear regression was used to investigate the relationship between weight misperception and weight loss, adjusting for baseline weight and BMI.

**Results:**

Those who had high levels of under-reporting at baseline had greater weight loss at three and 12 months compared with those who under-reported to some degree, and those over-reporting their weight. A significant association was found between weight misperception and weight loss at the three and the 12 month time points. Baseline weight was not associated with weight loss.

**Conclusions:**

Weight misperception should be acknowledged as a factor to be addressed when screening and identifying individuals at risk for diabetes. Screening and giving feedback is important in terms of awareness of participants’ actual weight status and may have an effect on program outcomes.

## Background

Waist circumference and weight are the main predictors of type 2 diabetes, and therefore weight loss and accurate weight perception are key parts in prevention.

It is widely acknowledged in the weight misperception literature that individuals tend to over-report their height and under-report their weight [1]. Various factors have been attributed to weight misperception such as age, gender, ethnicity, socio-economic status, being overweight or obese and a social desirability for tallness and thinness [1-3].

Under-reporting weight in overweight or obese individuals has been linked with reduced perception of health risks [4] and of the advantages of weight loss [5], and also less physical activity and attempts at weight loss [6]. However, when overweight perception is corrected positive associations between weight perception and weight loss have been identified [7-10].

Weight misperception may have an impact on perceived risk and susceptibility for chronic diseases. Little has been reported on the long term effects of this misperception in chronic disease interventions, particularly in field of diabetes prevention. If individuals misperceive their weight status and therefore disease risk, the effectiveness of intervention screening and program uptake may be reduced.

The aim of this paper was to investigate the association between weight misperception and weight loss during an Australian diabetes prevention program conducted with moderate to high risk individuals [11]. We hypothesise that there would be a positive relationship between weight misperception and weight loss at three and 12 months post intervention. The underlying assumption for this hypothesis was that individuals who have high levels of weight misperception are likely to be alarmed and more motivated into taking action upon realisation of actual weight status.

## Methods

The methods, recruitment process and results for the Greater Green Triangle Diabetes Prevention Project (GGT DPP) conducted in south east Australia between 2004-2006 have been previously been described [11]. The GGT DPP was a diet and lifestyle intervention in obese and overweight subjects at moderate to high risk of developing type 2 diabetes. Briefly, recruitment occurred through opportunistic screening by study nurses of rural individuals aged 40-75 years presenting at local general practices. Individuals who appeared to be over the age of 40 and who were considered overweight were approached during recruitment. A self-administered risk screening tool (Finnish Diabetes Risk Score, FINDRISC) was used to identify individuals at moderate to high risk of developing type 2 diabetes [12].

During recruitment individuals were asked to complete self-reported anthropometric information on the FINDRISC form which included a height, weight and waist circumference category [13]. Participating individuals attended a baseline testing session within three weeks of being screened, although most were tested on the same day as providing self-reported anthropometric information. Objectively measured weight was among the anthropometric measures taken by specially trained study nurses using the European Health Risk Monitoring protocol [14]. Participants attended a further three and 12 month testing session post-intervention where weight was measured amongst other anthropometric, biochemical and behavioural measures. Weight was measured using the same standardised mechanical column beam balance scales (Seca) at the three time points of testing.

### Analysis

A retrospective post-hoc analysis was undertaken with data collected during the implementation of the GGT DPP. Weight misperception was calculated as the difference between participants’ self-reported weight before baseline testing and the actual weight recorded at baseline testing. This variable was converted to per cent values. The weight recorded at baseline was measured in kg to one decimal place and was used to calculate BMI (kg/m^2^). The weight loss variables (i.e. three months and 12 months) were calculated as the difference between the clinically recorded weights relative to the weight at baseline and were converted to per cent values.

Linear regression (‘Enter’ method) was used to investigate the relationships between the dependent variables of percentage weight change at three and 12 months, and the explanatory variables of weight misperception, baseline weight and BMI. Weight misperception was included in all models with baseline weight and BMI included separately. These analyses were carried out on the pooled sample of males and females, and also stratified by gender. Linear regression was also used to assess the influence of age and years of formal education on weight misperception. Out of n = 311 eligible participants, n = 251 are included for these analyses at baseline because participants from one site (n = 60) were excluded as only objective measurements were recorded. At three months there were n = 201 participants, and at 12 months there were n = 208 participants included in the analyses.

### Ethics approval

Written informed consent was obtained from the GGT DPP participants for the publication of this report. This study was approved by the Flinders University Clinical Research Committee (reference number 105/034).

## Results

There were a total of n = 62 males and n = 189 females, the mean age for males was 57 years and the mean age for females was 56 years, the majority were married (males 74.2%, females 70.4%), and 63.3% of males and 49.2% of females were employed. Males had a mean 11.7 years of formal education compared to females 11.9 years. Mean weight for the pooled sample at baseline was 92.6 kg and mean weight loss at 3 months was 2.53 kg and mean weight loss at 12 months was 2.45 kg.

Weight misperception by categories of under or over-reporting and associated weight loss at three and 12 month follow up are presented in Table 1. Those who had a high level of under-reporting at baseline (>5%) had greater weight loss at three and 12 months (-3.52% and -3.70% respectively) compared with those who under-reported to some degree or over-reported their weight.

**Table 1:** Weight loss at 3 and 12 months by weight misperception categories

		**3 month follow up**			**12 month follow up**		
	**n**	**n**	**Weight % Δ 3 months (SE)**	**Weight kg Δ 3 months (SE)**	**n**	**Weight % Δ 12 months (SE)**	**Weight kg Δ 12 months (SE)**
Weight misperception category							
Under-report >5%	70	58	-3.5 (0.5)	-3.5 (0.5)	59	-3.7 (0.6)	-3.6 (0.6)
Under-report 2%-4.99%	72	59	-2.6 (0.5)	-2.8 (0.4)	60	-3.0 (0.8)	-2.9 (0.8)
Under or over report -1.99% - 1.99%	87	67	-1.2 (0.4)	-1.6 (0.3)	71	-1.7 (0.65)	-1.7 (0.6)
Over-report >2%	22	18	-2.3 (0.7)	-2.3 (0.5)	18	-0.5 (1.24)	-0.04 (1.28)
Total	251	201	-2.4 (0.3)	-2.5 (0.2)	208	-2.6 (0.4)	-2.5 (0.4)

Δ = change, kg = kilograms, SE = standard error.

There was a significant relationship between weight misperception and weight loss both at three and 12 months (B = 0.205 and B = 0.220 respectively) for both genders combined (Table 2). The same positive relationship was found for males and females, but was only significant in the latter. This was possibly because of the relatively low number of males in the sample (data not shown).

**Table 2:** Regression models examining weight misperception and weight loss at 3 and 12 months

		**Weight loss after 3 months**				**Weight loss after 12 months**			
		**Constant**	**B**	**p-value**	**Adj r**^**2**^	**Constant**	**B**	**p-value**	**Adj r**^**2**^
Model 1	Weight Misperception (%)	-1.790	0.205	<0.001	0.068	-1.94	0.220	0.006	0.032
Model 2	Weight Misperception (%)	-2.35	0.210	<0.001	0.065	0.378	0.208	0.01	0.033
	Baseline BMI (kg/m^2^)		0.017	0.679			-0.07	0.247	
Model 3	Weight Misperception (%)	-2.90	0.213	<0.001	0.068	-0.21	0.211	0.009	0.031
	Baseline Weight (kg)		0.012	0.368			-0.019	0.365	

Δ = change, kg = kilograms, BMI = Body Mass Index, Adj = Adjusted.

The variables baseline BMI and baseline weight were not found to be statistically significant, indicating that the relationship between weight misperception and weight loss at the two time periods were independent of baseline weight (Table 2). That is neither baseline weight nor BMI were significant predictors of weight loss. Approximately 7% of the variation of the weight loss at three months and 3% at 12 months were explained by weight misperception.

Age and years of formal education were non-significant explanatory variables of weight loss at three months (age: B = 0.038, p = 0.162; formal education: B = 0.007, p = 0.922) and 12 months (age, B = 0.034, p = 0.146; formal education: B = 0.006, p = 0.922) when included in multiple regression models with weight misperception. Weight misperception was significantly associated with weight loss at three months (B = 0.190, p < 0.001 in the model including age and B = 0.205, p < 0.001 in the model including formal education) and 12 months (B = 0.158, p < 0.001 in the model including age and B = 0.173, p < 0.001 in the model including formal education).

## Discussion

This study identified weight misperception as a variable associated with weight loss in a diabetes prevention program over time. This association existed independently of obesity level at baseline, indicating that misperception has the same effect in overweight and obese individuals. Furthermore, those who had high levels of under-reporting weight at baseline had greater weight loss compared with those who either under-reported to some degree or over-reported their weight.

We believe that individuals who have high levels of weight misperception (under-reporters) are likely to be alarmed into taking action and more motivated to lose weight compared with those who over-report or have accurate weight assessments. This finding reflects the social cognitive and self-regulation theories of health behaviour change [15-18], where motivation is formed through the development of outcome expectations, self-efficacy and risk perception [17,19,20]. According to these theories correcting weight misperception may be considered as a precursor or additional element which contributes to building risk perception, like the psychological and behavioural changes that have been shown to predict bio physiological outcomes in this diabetes prevention program [21].

There is a paucity of literature investigating the association between weight misperception and observed weight loss in prevention studies. It is possible that other predictors for diabetes, such as family history and experience of chronic disease, are stronger contributors for building risk perception than weight perception. Harwell et al. (2001) found that rural survey participants who were obese and had a family history of diabetes were more likely to consider themselves at risk for diabetes [22]. Dorman et al. (2012) found that those with increased familial risk for diabetes, CHD and stroke had high levels of perceived risk and worry about diabetes [23]. In contrast, Adriannse et al (2003) found that participants in a diabetes screening program with diabetes risk factors such as having a family history, obesity and hypertension, did not perceive themselves to be at increased risk for the disease [24]. The findings from this study emphasises the importance of population recognition and awareness of weight as one of the main contributors of risk for developing diabetes.

Weight misperception was an important explanatory variable indicating that the risk screening and giving feedback are essential components of the program. However weight misperception by itself has limited value for predicting percentage weight loss after three months and 12 months, as the intervention was the main contributor to weight loss. Further research is needed to confirm the predictive value of weight misperception towards building up risk perception and outcome expectations and their relationship to weight loss.

### Strengths and limitations

The lower proportion of males in the sample could be viewed as a limitation with potential implications for internal validity through selection bias. By carrying out an analysis stratified by gender it was found that the direction of the regression coefficient was the same for both genders despite being non-significant for males. This study was a post-hoc retrospective analysis of a prospective cohort so there are limitations when undertaking analysis of this kind. This includes being unable to assess other potential confounding factors which could change the associations found.

These results can only be generalized to intervention populations recruited from general practitioner clinics for diabetes prevention programs given the non-random selection of participants from the clinic waiting rooms. It is known that those recruited in interventions already have some motivation and intention for behaviour change [25]. These results, however, do provide important information for planning and implementation of lifestyle interventions.

## Conclusions

The results from this study suggest the importance of population screening and creating obesity awareness for diabetes risk. We suggest that weight misperception should be addressed during the screening process for diabetes prevention programs in order to better develop risk perception, build perceived susceptibility, and understand the seriousness of the disease risk.

## Competing interests

The authors declare that they have no competing interests.

## Authors’ contributions

AH conceived and designed the study. TL, EDJ and JAD were involved in acquisition of data. VV with AH, TL, EV and EDJ analysed and interpreted the data. AH drafted the manuscript and was responsible for its revisions. VV, TL, EV, EDJ and JAD contributed to specific sections in the manuscript. JAD and was the chief investigator for the GGT DPP. All authors read and approved the final manuscript.

## Pre-publication history

The pre-publication history for this paper can be accessed here:

http://www.biomedcentral.com/1471-2458/14/93/prepub
